# Intensified Influenza Virus Production in Suspension HEK293SF Cell Cultures Operated in Fed-Batch or Perfusion with Continuous Harvest

**DOI:** 10.3390/vaccines11121819

**Published:** 2023-12-05

**Authors:** Cristina A. T. Silva, Amine A. Kamen, Olivier Henry

**Affiliations:** 1 Department of Chemical Engineering, Polytechnique Montréal, Montreal, QC H3T 1J4, Canada; 2 Department of Bioengineering, McGill University, Montreal, QC H3A 0E9, Canada; amine.kamen@mcgill.ca

**Keywords:** influenza vaccine, process intensification, perfusion, fed-batch, suspension cells, high cell density, HEK293, continuous harvest

## Abstract

Major efforts in the intensification of cell culture-based viral vaccine manufacturing focus on the development of high-cell-density (HCD) processes, often operated in perfusion. While perfusion operations allow for higher viable cell densities and volumetric productivities, the high perfusion rates (PR) normally adopted—typically between 2 and 4 vessel volumes per day (VVD)—dramatically increase media consumption, resulting in a higher burden on the cell retention device and raising challenges for the handling and disposal of high volumes of media. In this study, we explore high inoculum fed-batch (HIFB) and low-PR perfusion operations to intensify a cell culture-based process for influenza virus production while minimizing media consumption. To reduce product retention time in the bioreactor, produced viral particles were continuously harvested using a tangential flow depth filtration (TFDF) system as a cell retention device and harvest unit. The feeding strategies developed—a hybrid fed-batch with continuous harvest and a low-PR perfusion—allowed for infections in the range of 8–10 × 10^6^ cells/mL while maintaining cell-specific productivity comparable to the batch control, resulting in a global increase in the process productivity. Overall, our work demonstrates that feeding strategies that minimize media consumption are suitable for large-scale influenza vaccine production.

## 1. Introduction

Millions of people are afflicted with the flu every year, a disease resulting from an infection with an influenza virus that causes more than half a million deaths around the world, particularly amongst the more vulnerable groups of the population [1]. As vaccination is the most efficient protective measure against the disease, seasonal vaccination campaigns require millions of doses to be produced in short timelines, and billions of doses would be necessary to respond to a possible pandemic scenario [2,3]. Our recent experience with the COVID-19 pandemic has shown that the development of different vaccine production platforms is essential to guarantee a worldwide supply of vaccines. In this scenario, cell culture-based processes emerge as a more flexible and responsive platform for influenza vaccine production and are a valuable alternative to traditional egg-based manufacturing systems [4]. Cell culture-based influenza vaccine manufacturing involves the production of whole viruses for inactivated vaccine formulations, which is achieved by cultivating mammalian or avian cells to sufficiently high cell densities and infecting those cells with the desired virus strain. When compared to traditional egg-based systems, cell culture processes offer various advantages: (i) cell culture processes can be easily scaled up, particularly when employing cells grown in suspension, with operations in tightly controlled bioreactors of more than 2000 L [5]; (ii) less risk of introducing antigenic modifications due to adaptation of virus for growth in eggs [6,7]; (iii) fast response to pandemic scenarios, as cell-based production can be accelerated by the use of high-cell-density (HCD) stocks and perfusion techniques [8,9]. Additionally, the development of single-use systems has increased the flexibility of cell culture manufacturing plants while reducing the upfront capital costs for building a new facility [10].

HEK293 cells are widely accepted as an expression platform and have been extensively employed for the production of recombinant proteins and viral vectors [11,12,13,14]. A growing number of therapeutic agents produced using these cells are currently on the market and have been approved for use by the FDA and the EMA [11], including the COVID-19 vaccine developed by the University of Oxford and AstraZeneca (ChAdOx1 nCoV-19) [15], highlighting the safety of this cell substrate. HEK293 cells have been successfully adapted for growth in suspension and are capable of growing to high cell densities in animal component-free media [12,16], characteristics that make them a valuable host for large-scale viral vaccine manufacturing. 

In order to be consolidated as a cost-effective vaccine manufacturing system, cell culture-based processes need to be intensified, essentially by addressing two main factors: (i) increasing the viable cell density (VCD) at the time of infection (TOI) while maintaining cells in a desired metabolic state, to increase final virus yield and avoid the so-called “cell density effect” (CDE)—characterized by a loss in cell-specific productivity (number of viruses produced per cell) when the infection occurs at higher cell densities [17,18,19]; (ii) decreasing the residence time of the viral particles in the bioreactor to limit product degradation [20]. In order to sustain high cell concentrations and therefore increase process productivity, fed-batch (addition of a concentrated feed media) and perfusion strategies (continuous exchange of media with cells kept inside of the reactor by a cell retention device) are usually employed [21,22,23]. However, as nutrient and by-product concentrations have been shown to impact virus production, feeding strategies must be designed to maintain the cells in a desired metabolic state by keeping key nutrients/metabolites within defined thresholds to avoid limitations/inhibitions [24,25,26]. Metabolic flux analysis performed in HEK293 cells showed that the CDE observed for HCD cultures could be overcome by keeping the metabolic profile of cells at similar levels to those of low-cell-density batch processes [18]. In that sense, the estimation of the average specific uptake/production rates for the main metabolites during the course of the process can be a simpler approach to evaluate the metabolic state of cells based on measurements of nutrient concentration and cell count and provide valuable information for the design and control of feeding strategies. 

Successful influenza virus production in HCD processes operated in perfusion is reported [20,27,28,29,30]. While perfusion operations allow for higher viable cell densities and productivities [21], the high perfusion rates (PR) normally adopted—typically between two and four vessel volumes per day (VVD)—raises challenges for the preparation, handling, and disposal of high volumes of fluid [21]. Additionally, due to the higher flux through the membrane, high perfusion rates can increase the risk of progressive membrane fouling as well as increase the shear stress to which the cells are submitted. High inoculum fed-batch (HIFB) processes can drastically reduce the volume of media consumed while sustaining relatively high viable cell densities [5,31,32] and are typically applied when the production is limited by nutrient depletion instead of by-product accumulation. The combination of HIFB processes with a perfusion-based continuous harvest approach serves to reduce the retention time of viral particles in the reactor while alleviating by-product accumulation and offers the possibility of working with integrated continuous/semi-continuous processes [33]. Membrane-based cell retention devices, such as the alternating tangential flow (ATF) and tangential flow filtration (TFF) modules, have been extensively applied in perfusion processes for virus production [27,28,34,35,36,37]. However, virus retention due to progressive membrane clogging has been reported for those modules for various membrane pore sizes [27,38]. Tangential flow depth filtration (TFDF) membranes combine the advantages of both tangential filtration and depth filtration—the latter being typically applied as an alternative to centrifugation for the harvesting step in virus production processes—allowing for the processing of HCD cultures with minimal membrane fouling and high product recovery [39,40,41]. Additionally, TFDF modules are scalable and single-use and are currently commercially available for up to 2000 L bioreactor scale.

In this study, two scalable HCD processes, which explore HIFB and low perfusion rate operations, are developed for influenza A virus (IAV) production in suspension HEK293SF cells. The perfusion-based operations are performed using a TFDF system as a cell retention device and harvest unit. Feeding strategies were designed and evaluated based on the assessment of the metabolic state of cells through the estimation of specific uptake/production rates for the main metabolites. 

## 2. Materials and Methods

### 2.1. Cell Line and Culture Conditions

Suspension HEK293SF cells were kindly provided by the National Research Council Canada and were cultivated at 37 °C and 5% CO_2_ in basal serum-free Xell HEK GM (Sartorius Stedim, Göttingen, Germany) medium supplemented with 6 mM L-Glutamine. Cells were maintained in 125 mL shake-tubes (25 mL working volume) using an orbital shaker at 135 rpm. Cell counting was performed using Vi-Cell XR Cell Viability analyser (Beckman Coulter, Pasadena, CA, USA). Analysis of main metabolite, glutamine (GLN), glutamate (GLU), glucose (GLUC), lactate (LAC), and ammonium (NH4) was performed using a blood analyzer BioProfile^®^ FLEX2 (Nova Biomedical, Waltham, MA, USA). Average specific uptake/production rates in a given interval for a metabolite *S* ($q_{s}$) were determined based on the following equations:

(1) $$q_{s}=\frac{\left(V_{n}*S_{n}-V_{n-1}*S_{n-1}\right)-V_{perf}*\left(S_{perf}-S_{ave}\right)-V_{feed}*S_{feed}}{\left(t_{n}-t_{n-1}\right)*XV_{ave}}$$

(2) $$S_{ave}=0.5*\left(S_{n-1}+S_{exp}\right)$$

(3) $$XV_{ave}=0.5*\left(V_{n}X_{n}+V_{n-1}X_{n-1}\right)$$

where $V_{n}$ is the culture volume at a given time ($t_{n})$, $S_{n}$ is the concentration of a metabolite S at $t_{n}$, $V_{perf}$ is the volume of perfused media in the given interval, $S_{perf}$ is the concentration of metabolite *S* in the perfusion media, $S_{ave}$ is the estimated average concentration of metabolite S in the reactor during the given interval, $S_{exp}$ is the expected concentration of the metabolite S if the media was exchanged semi-continuously (for semi-continuous cultures, $S_{ave}=S_{n-1}$), $V_{feed}$ is the volume of concentrated feed added in the given interval, $S_{feed}$ is the concentration of metabolite S in the concentrated feeding media, $XV_{ave}$ is the estimated average number of cells in the given interval, and $X_{n}$ is the viable cell density at $t_{n}$.

Negative values of $q_{s}$ indicate consumption of the nutrient/metabolite, while positive values indicate its production. However, to facilitate the discussions, comparisons between different consumption rates are made considering the absolute values. 

The molar lactate-to-glucose $\left(Y_{LAC/GLUC}\right)$ and ammonium-to-glutamine ratios $\left(Y_{NH4/GLN}\right)$ were calculated as follows:

(4) $$Y_{LAC/GLUC}=\frac{q_{LAC}}{-q_{GLUC}}$$

(5) $$Y_{NH4/GLN}=\frac{q_{NH4}}{-q_{GLN}}$$

### 2.2. Virus Strains and Infection Conditions

Viral stocks of influenza A/Puerto-Rico/8/34 (H1N1), originally derived from ATCC’s Global Bioresource Center, were adapted by serial passaging and produced in HEK293SF cells [42]. Infections were performed without media exchange, at a multiplicity of infection (MOI) of 10^−3^ with the addition of TPCK trypsin (Sigma, St. Louis, MO, USA) at a final concentration of 1 µg/mL. After infection, the temperature was shifted to 35 °C. 

### 2.3. Feeding Strategies and Process Operation

Two different feeding strategies for HCD processes, aiming to minimize the volume of media consumed, were developed in small-scale experiments and scaled up to a 3 L benchtop bioreactor (see following sections for experimental setup). For the HIFB operation, cultures were inoculated at 2 × 10^6^ cells/mL in basal media at 80% of the final working volume. Daily addition of concentrated, chemically defined and serum-free media CellBoost5 (Cytiva, Marlborough, MA, USA) supplemented with 30 mM L-glutamine was performed. The volume of concentrated feeding media to be added varied from 5 to 15% (*v*/*v*) of the culture volume and was determined based on the growth rate and the values for *q_GLN_* (Equation (1)) for the previous sampling interval. Based on these values and using basic mass balance equations for viable cell density and nutrient concentration, a prediction for glutamine consumption for the next interval was made, and a corresponding volume of feed was added to replenish consumed glutamine and maintain its concentration above limiting values. After infection, performed at a cell density between 8 and 9 × 10^6^ cells/mL, a continuous harvest of the produced virus was performed at a PR of 0.5 VVD with perfusion media (basal media supplemented with 10% *v*/*v* CellBoost5 and 8 mM L-glutamine). For the perfusion operations, cultures were inoculated at 1.5 × 10^6^ cells/mL in basal media. A PR of 0.5 VVD was used for the small-scale experiments throughout the process using a perfusion media composed of basal media supplemented with 10% *v*/*v* CellBoost5 and 8 mM L-glutamine. On bioreactor operations, the PR was adjusted based on cell metabolism to keep nutrients above minimum levels and to limit by-product accumulation, varying between 0.4 and 0.8 VVD. The perfusion rate and media supplementation were defined based on average values for growth rate and *q_s_* (Equation (1)) obtained in preliminary experiments and basic mass balance equations for cell growth and nutrient consumption. 

### 2.4. Small-Scale Experiments

Small-scale experiments were conducted in 50 mL shake-tubes (TPP Techno Plastic Products AG, Trasadingen, Switzerland) agitated at 180 rpm with an inclination of 45⁰ in an orbital shaker incubator with an orbital throw of 25 mm. Cultures were maintained at an approximately constant working volume of 10 mL (some variations occurred due to culture sampling and addition of feed). Cells were infected between 2 and 10 × 10^6^ cells/mL, depending on the tested strategy. In order to simulate both the perfusion and the continuous harvest operations, shake-tubes were operated in a pseudo-perfusion mode whereby, on a daily basis, tubes were centrifuged for 3 min at 300× *g* and a previously determined volume of media, equivalent to 0.5 VVD, was removed and replenished with fresh perfusion media. All small-scale experiments were performed in duplicates.

### 2.5. Bioreactor Set-Up and Operation

A 3 L benchtop bioreactor (Applikon Biotechnology, Delft, The Netherlands) equipped with two marine impellers was used for all of the strategies tested. The bioreactor was maintained at 37 °C, pH 7.1, and stirring speed of 100 rpm. The temperature was shifted to 35 °C for the infection phase. Dissolved oxygen was controlled at 40% of air saturation via surface aeration and pure oxygen sparging through a micro-sparger. The bioreactor was equipped with a capacitance probe (Aber Instruments, Aberystwyth, UK) for biomass monitoring. A 30 cm^2^ (pore size 2.0–5.0 µm) TFDF membrane (Repligen Corporation, Waltham, MA, USA) operated by the KML-100 system (Repligen Corporation, USA) was employed as cell retention device for perfusion and continuous harvest operations. The recirculation rate was maintained at 1 L/min, measured by an integrated flowmeter. Continuous addition of perfusion media was regulated by a weight control system to maintain bioreactor level according to the permeate flow rate. After infection, the permeate material was collected in ice-cooled bottles, which were exchanged at every sample point (permeate pool). At the time of harvest (TOH), determined based on the capacitance signal and on the culture viability, the bioreactors were harvested through the cell retention device. The final harvest comprised a primary concentration step, followed by a diafiltration and a final concentration, which resulted in a 20% increase in the final volume. To avoid membrane clogging, the recirculation rate was increased to 2 L/min prior to harvesting.

### 2.6. Virus Quantification and Productivity Evaluation

Hemagglutinin (HA) content was evaluated using hemagglutination assay based on the virus’s capacity to reticulate red blood cells. Briefly, serial-diluted samples were incubated with purified chicken erythrocytes (Innovative Research, Novi, MI, USA) at room temperature for 2–12 h. Plates were read based on the presence of a pellet of sedimented red blood cells. Quantification of infectious virus particles (IVP) was performed using TCID50. Briefly, adherent MDCK cells were cultured in VP-SFM culture medium (Gibco, New York, NY, USA) supplemented with 1% Pen/Strep (Gibco, USA) in a 96-well plate at a seeding density of 0.2 × 10^6^ cells/well. After 24 h, the culture media was exchanged for trypsin-containing medium (1 µg/mL) and infected with 5-fold serial dilutions of infectious culture supernatant. Plates were incubated for 4–7 days at 37 °C and evaluated for the presence/absence of cytopathic effect. TCID titers were calculated following the Reed–Muench Method [43]. 

Three productivity parameters were selected to compare the different production processes and were calculated based on the total virus yield at a given time $\left(Y_{T}\right)$ using the hemagglutination titers:

(6) $$Y_{T}=\left[C_{R}+\sum_{0}^{t}{(C}_{P}*PR)\right]*V_{R}$$

where $C_{R}$ is the concentration of virus in the reactor at a given time, $C_{P}$ is the concentration of virus in the permeate pool, PR is the perfusion rate, and $V_{R}$ is the reactor working volume. 

The cell-specific virus yield (*CSVY*), the space–time yield (*STY*), and the yield on media (*Y/M*) were calculated as follows:

(7) $$CSVY=\frac{Y_{\;T}}{X_{T}*V_{R}}$$

(8) $$STY=\frac{Y_{T}}{V_{R}*t_{T}}$$

(9) $$Y/M=\frac{Y_{\;T}}{V_{T}}$$

where $X_{T}$ is the maximum total cell concentration, $t_{T}$ is the total culture duration, and $V_{T}$ is the total volume of media consumed.

## 3. Results

### 3.1. Small-Scale Characterization of the CDE

**Figure 1:**
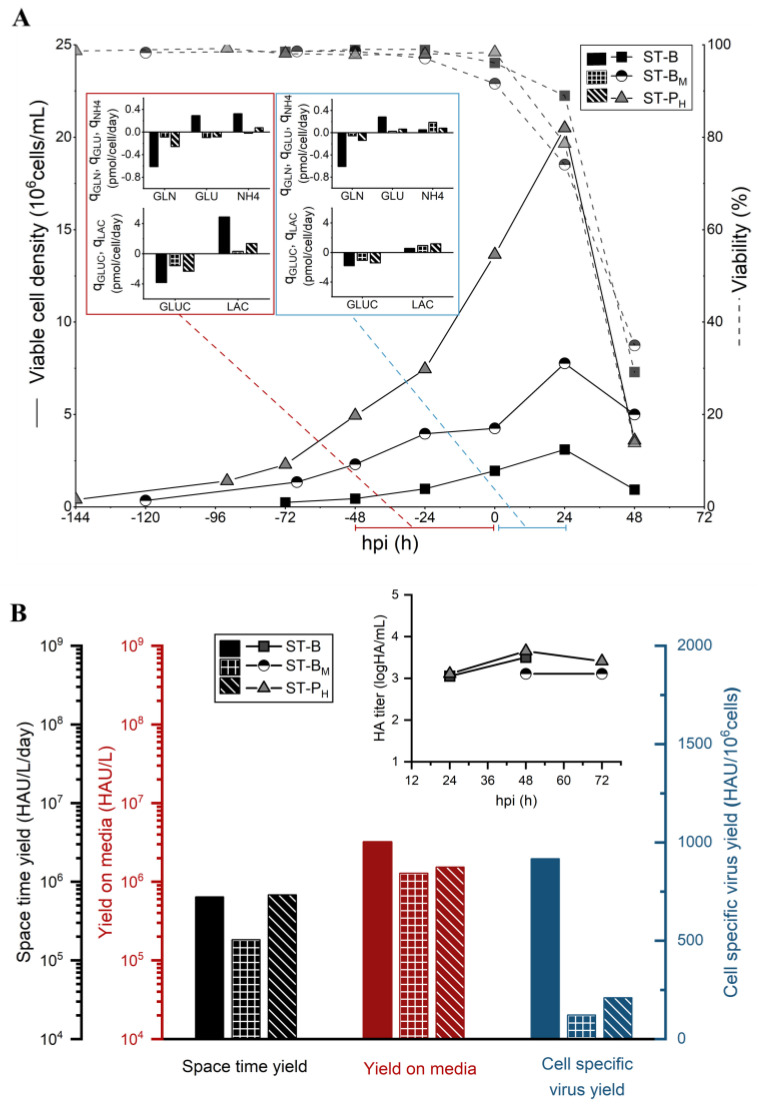
(**A**) Comparison of cell growth, viability, and average specific consumption/production rates (*q_S_*) of main metabolites and (**B**) productivity of 50 mL shake-tube cultures of HEK293SF cells for influenza virus production, consisting of low-cell-density batch (ST-B, infected at 2 × 10^6^ cells/mL), medium-cell-density batch (ST-B_M_, infected at 4 × 10^6^ cells/mL), and high-cell-density pseudo-perfusion, 0.5 VVD, (ST-P_H_, infected at 13 × 10^6^ cells/mL). Insets show the average cell-specific rates before and after infection.

In order to characterize the effect of potential nutrient limitations in HCD processes for IAV production in HEK293 cells, three operation strategies were tested in small-scale experiments: a low-cell-density batch control (ST-B) infected at 2 × 10^6^ cells/mL, a medium-cell-density batch (ST-B_M_) infected at 4 × 10^6^ cells/mL, and a high-cell-density pseudo-perfusion (ST-P_H_) with a fixed PR of 0.5 VVD (non-supplemented basal media) infected at 13 × 10^6^ cells/mL. All of the experimental conditions tested are summarized in Table 1. During the 24 h that followed the infection, cells continued growing for all three conditions tested, reaching 3.2 × 10^6^ cells/mL for the ST-B culture, 8.1 × 10^6^ cells/mL for ST-B_M_, and 20.5 × 10^6^ cells/mL for ST-P_H_ (Figure 1A). As expected, a clear drop in *CSVY* was observed for both ST-B_M_ and ST-P_H_ strategies when compared to the low-cell-density batch cultures (Figure 1B). The drop in *CSVY* was reflected in a lower *Y/M* and a decrease, or no significant increase, in the *STY*. Average cell-specific uptake/production rates for the main metabolites (*q_S_*) were assessed before and after infection based on mass balance equations. The average specific uptake rate of glutamine (*q_GLN_*), as well as the specific production of ammonium (*q_NH4_*), was significantly lower both before and after infection for the medium and high-cell-density strategies (Figure 1A). This decrease in the uptake rate is most likely linked to the low residual concentrations of this nutrient in the culture media [44], which was below 1 mM at the TOI for both cultures (Figure S1). Interestingly, although residual glutamine concentrations were lower at 24 hpi (hours post-infection) for both ST-B_M_ and ST-P_H_ cultures—below 0.5 mM compared to 2.4 mM for the ST-B cultures (Figure S1)—their respective average *Y_NH4/GLN_* for the 24 h following the infection was higher (Table 2), indicating less efficient glutamine metabolism after infection. While the average specific uptake of glucose (*q_GLUC_*) was also lower both before and after infection for the medium- and high-cell-density cultures, the difference between those and that of the ST-B batch control was less pronounced, even though the residual concentrations of this nutrient were significantly different—6.5 mM and 9.5 mM, respectively, for the ST-B_M_ and ST-P_H_ cultures, and 30 mM for ST-B. For this reason, the feeding strategies for the HCD processes were mainly designed based on specific glutamine consumption.

### 3.2. Small-Scale Development of HCD Feeding Strategies

**Figure 2:**
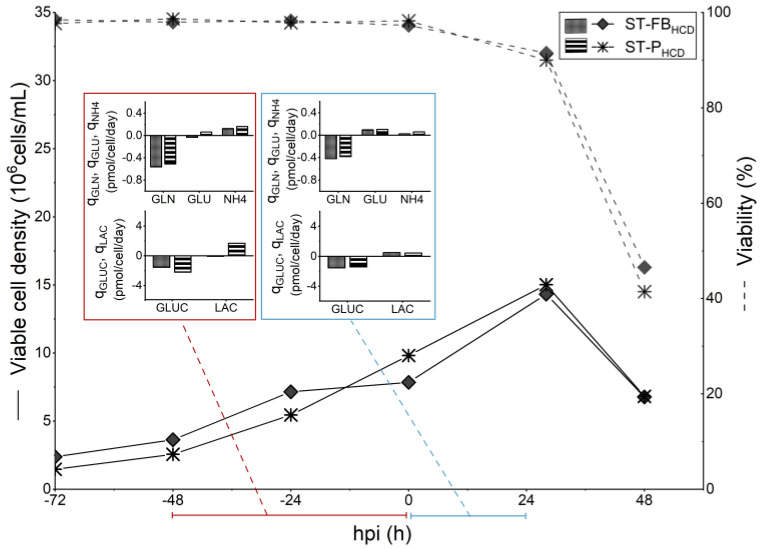
Comparison of cell growth, viability, and average specific consumption/production rates (*q_S_*) of main metabolites for 50 mL shake-tube cultures of HEK293SF cells for influenza virus production, consisting of HCD hybrid fed-batch/pseudo-perfusion (ST-FB_HCD_, infected at 8.5 × 10^6^ cells/mL) and HCD pseudo-perfusion (ST-P_HCD_, infected at 10 × 10^6^ cells/mL).

Based on the average consumption/production rates observed for the low-cell-density batch process, two HCD feeding strategies, targeting to minimize the volume of media consumed while keeping the uptake/production profiles similar to the batch control, were designed and evaluated in shake-tubes: a hybrid fed-batch with continuous harvest (ST-FB_HCD_) and a perfusion strategy (ST-P_HCD_), both described in detail in Table 1. In order to supply other nutrients and metabolites that were not quantified, a concentrated feeding media, Cell Boost 5, was chosen for the bolus feeding additions in the fed-batch strategy and to supplement the perfusion media for the perfusion-based strategy. The amount of concentrated feed to be added was determined based on glutamine consumption, following the assumption that those non-quantified metabolites would be consumed at a similar rate. ST-FB_HCD_ and ST-P_HCD_ cultures reached, on average, 14.3 × 10^6^ cells/mL and 15 × 10^6^ cells/mL, respectively, at 24 hpi (Figure 2). Unlike what was observed for the non-optimized cultures (ST-B_M_ and ST-P_H_), the average *q_GLN_* was comparable between the HCD cultures and the low-cell-density batch control (ST-B), both before and after infection (Figure 2). While *Y_NH4/GLN_* was higher before infection for the ST-B culture, indicating a less efficient glutamine metabolism for the cultures operated in batch, cultures in all three conditions reached similar levels for this ratio in the 24 h that followed the infection (Table 2). Although average *q_GLUC_* was less important before infection for both HCD cultures, *q_LAC_* was also smaller, which was reflected in lower values of *Y_LAC/GLUC_* for those cultures (Table 2), indicating a more efficient use of the sugar. It is worth noting that glucose concentrations at the TOI were above 15 mM for both ST-P_HCD_ and ST-FB_HCD_ (Figure S2), showing that no limitations of this nutrient occurred. After infection, *q_GLUC_* and *q_LAC_* attained similar levels for both the HCD cultures (ST-P_HCD_ and ST-FB_HCD_) and the ST-B control.

**Figure 3:**
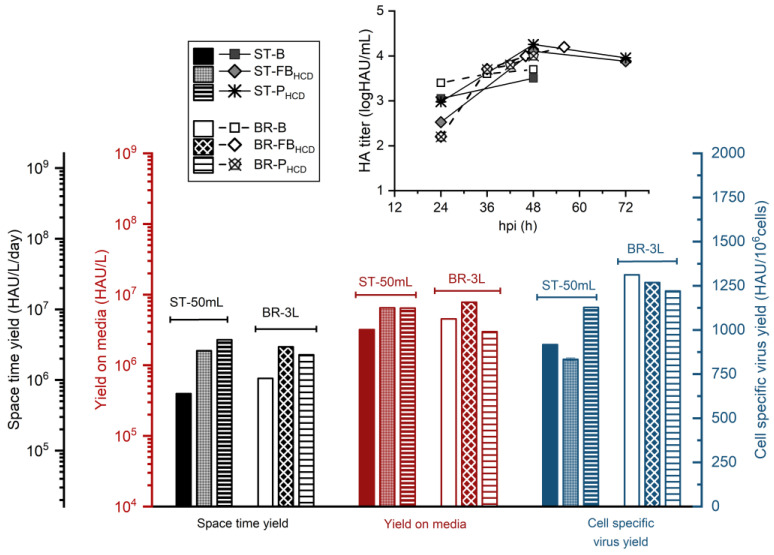
Virus titers and productivity of 50 mL shake-tube (ST) and 3 L bioreactor (BR) cultures of HEK293SF cells for influenza virus production, operated in low-cell-density batch (B, infected at 2 × 10^6^ cells/mL), HCD hybrid fed-batch/perfusion (FB_HCD_, infected at 8.5 × 10^6^ cells/mL), and HCD perfusion (P_HCD_, infected between 8.5–10 × 10^6^ cells/mL). Perfusion operations in shake-tube cultures were performed semi-continuously (pseudo-perfusion).

With respect to virus production, HA content at TOH (48 hpi) was, respectively, 0.6- and 0.8-log higher for the ST-FB_HCD_ and ST-P_HCD_ cultures (Table 1). The *CSVY* was comparable for all three conditions (Figure 3), demonstrating that the CDE was successfully overcome. Even though the developed HCD feeding strategies resulted in a higher volume of media consumed, the *Y/M* was 2-fold higher, and the *STY* was, respectively, 4- and 6-fold higher for the HCD fed-batch and perfusion strategies, in comparison to the batch control. In order to better characterize the produced material, the infectious titer at TOH was determined for the different conditions tested (Table 1). The infectious titer at TOH was, respectively, 6- and 7-fold higher for the ST-FB_HCD_ and ST-P_HCD_ cultures when compared to the batch control, which suggests that the material produced by the HCD cultures kept its infectivity.

### 3.3. Scale Up to 3 L STR Bioreactor

**Figure 4:**
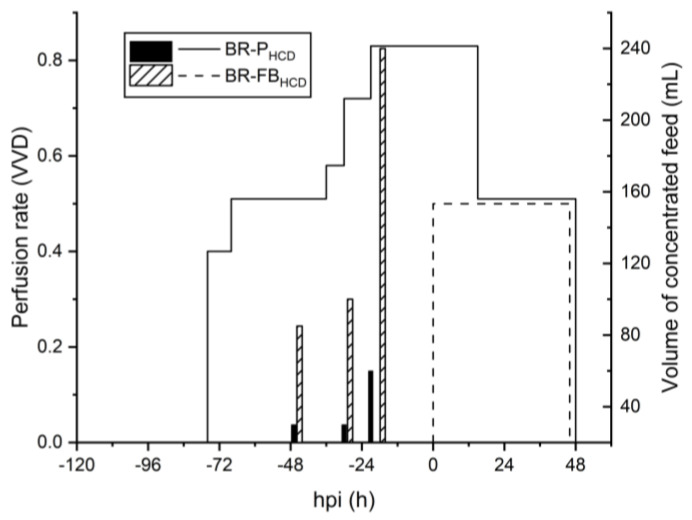
Perfusion rate (lines) and volume of concentrated feed (bars) for 3 L bioreactor cultures operated in perfusion (BR-P_HCD_) and hybrid fed-batch/perfusion (BR-FB_HCD_) for influenza virus production in suspension HEK293SF cells.

**Figure 5:**
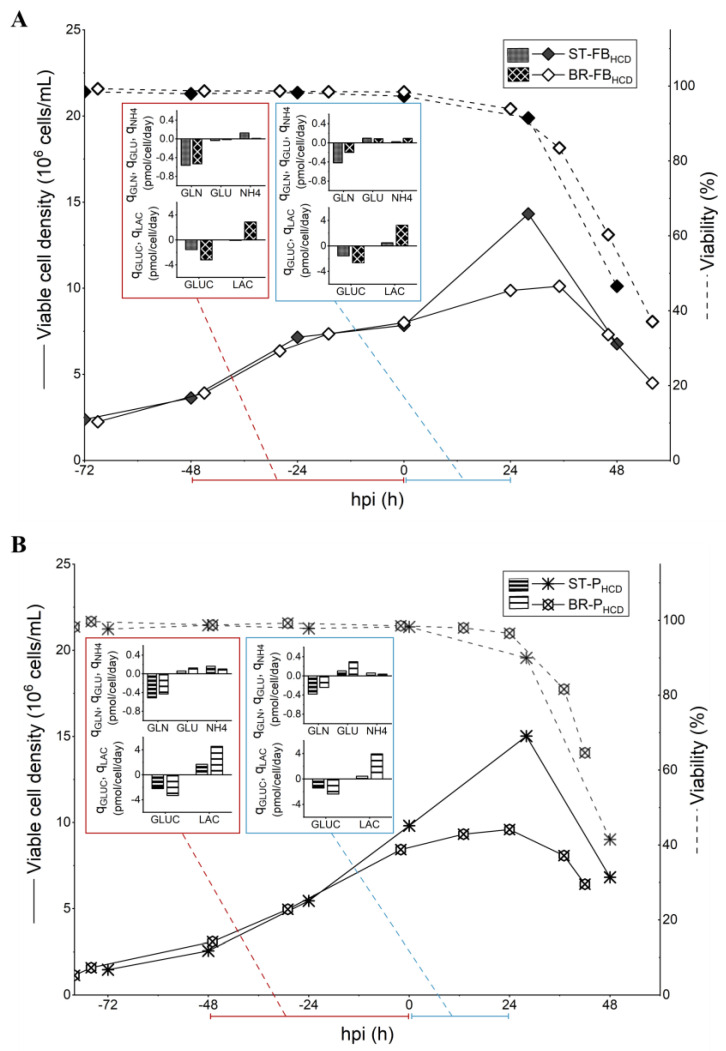
Comparison of cell growth, viability, and average specific consumption/production rates (*q_S_*) of main metabolites for 50 mL shake-tube (ST) and 3 L STR bioreactor (BR) cultures of HEK293SF cells producing influenza virus, operated in (**A**) fed-batch (FB_HCD_) and (**B**) perfusion (P_HCD_). Perfusion operations in shake-tube cultures were performed semi-continuously (pseudo-perfusion).

To evaluate the scalability of the designed strategies, two perfusion-based runs were performed in a 3 L STR bioreactor, using a TFDF system as the cell retention device. A scale-up of the batch run was also performed for comparison, and the TFDF system was employed for the harvest step. The feeding profile for both HCD runs is presented in Figure 4. For the hybrid fed-batch strategy (BR-FB_HCD_), the bioreactor was inoculated at 2 × 10^6^ cells/mL. The addition of concentrated feeding media was performed approximately every 16 h during the growth phase (Figure 4), and the volume added was determined based on specific consumption/production rates for glutamine, as defined in the small-scale experiments. After infection, the culture was shifted to perfusion mode with a fixed PR of 0.5 VVD to continuously harvest the produced virus. Since the perfusion was kept off during the 4 h that followed the infection—so as to ensure virus entry into the cells—the equivalent volume of media for that time period was exchanged right before the infection. Cells were infected at 8.5 × 10^6^ cells/mL and, differently from what was observed for the same strategy performed in shake-tubes, the cell-specific growth rate decreased drastically after infection, with cells reaching a maximum concentration of 10.1 × 10^6^ cells/mL at 35 hpi (Figure 5A). Nevertheless, the viability profile and the growth profile before infection were comparable for both scales. Metabolic profiles greatly varied for the two different production scales. While glutamine consumption was similar during the growth phase, bioreactor cultures presented higher average *q_GLUC_* and *q_LAC_* before and after the infection. Additionally, the glucose metabolism was less efficient for the BR-FB_HCD_ culture in comparison to the ST-FB_HCD_ cultures, with values of *Y_LAC/GLUC_* 13-fold and 4-fold higher for the first, respectively, before and after infection. That led to lactate accumulation up to 55 mM right before infection for the bioreactor culture, compared to 10 mM for the equivalent shake-tube cultures (Figure S3). This discrepancy in the metabolism between both production scales could be related to bioreactor operation parameters, particularly DO and pH control [45]. Despite the differences in growth and metabolism, HA content from the bioreactor was similar to the small-scale model throughout the run (Figure 3), and although it was slightly lower for the BR-FB_HCD_, the overall difference was within the assay error (0.15 logHAU/mL). Consequently, values for *STY* and *Y/M* were slightly lower for the bioreactor run but remained comparable. On the other hand, the *CSVY* was significantly higher for the bioreactor culture—1268 HAU/10^6^ cells for the BR-FB_HCD_ compared to 834 HAU/10^6^ cells for ST-FB_HCD_—as the HA content was comparable throughout the run between both scales even though the maximum cell density reached was lower for the bioreactor. However, the infectious titer at TOH was lower for the BR-FB_HCD_ (Table 1). While this decrease in infectivity could be related to the accumulation of lactate in the bioreactor, it could also be partially related to the difference between the semi-continuous operation in the shake-tubes and the continuous harvest from the bioreactor.

For the intensified perfusion strategy (BR-P_HCD_), the bioreactor was inoculated at 1.5 × 10^6^ cells/mL. Since previous attempts to scale up this strategy showed that differences in the metabolism and nutrient supply, mainly regarding the glucose metabolism, between the semi-continuous small-scale model and the continuous perfusion bioreactor resulted in nutrient limitations and lower titers for the latter, a different strategy was applied for the feeding and the PR control. A stepwise increase of 20% in the PR was performed based on nutrient concentration and specific consumption/production rates for the main metabolites. During the course of the run, the PR varied from 0.4 to 0.8 VVD (Figure 4), with an average PR of 0.56 VVD. Concentrated feeding was added to the bioreactor as a separated stream—instead of directly supplemented in the perfusion media—and the volume to be added was defined based on specific glutamine and glucose consumption. The bioreactor was infected at 8.6 × 10^6^ cells/mL and, similarly to what was observed for the hybrid fed-batch strategy, cell growth drastically decreased after infection, with cells reaching a maximum cell concentration of 10 × 10^6^ cells/mL—compared to 15 × 10^6^ cells/mL for the same strategy performed in shake-tubes (Figure 5B). The BR-P_HCD_ culture presented, respectively, higher specific uptake and production rates for glucose and lactate both before and after the infection when compared to the ST-P_HCD_ cultures (Figure 5B). That led to a lactate concentration of 38 mM right before infection, compared to 15 mM for the semi-continuous shake-tube cultures (Figure S3). Regarding virus production, HA content was slightly lower for the bioreactor run in comparison to the same strategy performed in shake-tubes (Figure 3), which resulted in an overall lower productivity for the BR-P_HCD_ culture (Table 1). The different feeding regimes for the PR resulted in a higher consumption of media for the bioreactor culture—4 × V_R_ for the BR-P_HCD_ compared to 2.9 × V_R_ for the ST-P_HCD_ cultures—which resulted in a 2-fold lower *Y/M* for the first. As observed for the hybrid fed-batch strategy, the *CSVY* was higher for the bioreactor culture, at 1220 HAU/10^6^ cells for the BR-P_HCD_ and 1129 HAU/10^6^ cells for the ST-P_HCD_, which can also be related to the lower maximum cell density reached in the bioreactor.

**Figure 6:**
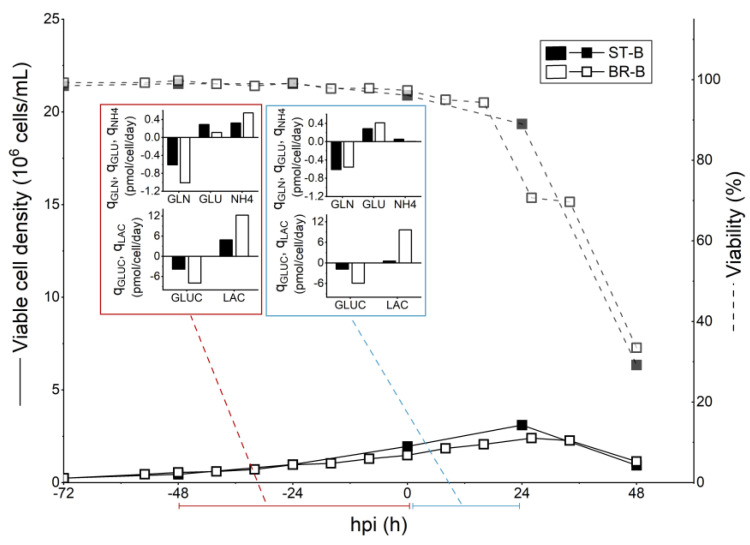
Comparison of cell growth, viability, and average specific consumption/production rates (*q_S_*) of main metabolites for 50 mL shake-tube (ST) and 3 L bioreactor (BR) cultures of HEK293SF cells producing influenza virus, operated in batch.

For scaling up the batch operation, the bioreactor was inoculated at 0.25 × 10^6^ cells/mL, and the infection was performed at 1.6 × 10^6^ cells/mL around 3 days later. Interestingly, the same reduction in cell growth after infection was observed, with a maximum VCD of 2.4 × 10^6^ cells/mL compared to 3.5 × 10^6^ cells/mL for the ST-B run (Figure 6). As the recirculation of the culture through the TFDF membrane was not started until the harvest, this decrease in the growth rate is most probably not related to shear stress in the membrane module but rather to the bioreactor operation and control. As was observed for the previously discussed bioreactor runs, specific glucose uptake and lactate production were higher for the bioreactor (BR-B) throughout the run when compared to the shake-tube cultures operated in batch (ST-B). Furthermore, even though the glucose concentration was 2-fold lower at infection for the BR-B (Figure S3), average specific lactate production in the 24 h that followed the infection was 15-fold higher for the BR-B culture and glucose uptake was 3-fold higher for the same base of comparison (Figure 6). These results indicate that the metabolism of cells in the bioreactor was much less efficient than that of cells cultured in shake-tubes, and as previously discussed, these differences are most likely linked to oxygen supply and/or pH control. Nonetheless, final HA content was higher for the bioreactor run at 3.7 logHAU/mL at TOH compared to 3.5 logHAU/mL at TOH for the ST-B culture (Table 1), which resulted in an overall higher productivity for the bioreactor (Figure 3). However, infectious virus titer at TOH was almost 2-fold lower for the BR-B run, and this decrease in infectivity could be related to the higher lactate concentration attained in the bioreactor, 50 mM in comparison to 19 mM for the shake-tube batch cultures (Figure S3).

Regarding the productivity of all three bioreactor runs, *CSVY* values were comparable between the HCD strategies and the low-cell-density batch—at 1311 HAU/10^6^ cells for BR-B, 1268 HAU/10^6^ cells for BR-FB_HCD_, and 1220 for BR-P_HCD_ (Table 1). *CSVY* values calculated based on the infectious virus titers (*CSVY_TCID_*) for the batch and perfusion bioreactors were also comparable, at 15 IVP/cell for BR-B and 14 IVP/cell for BR-P_HCD_ (Table 1). Because of the higher consumption of media during the perfusion run, the *Y/M* was 1.5-fold lower for the BR-P_HCD_ culture in comparison to the batch control. On the other hand, the *Y/M* was 1.7-fold higher for BR-FB_HCD_ when compared to the batch control. Values of *STY* were, respectively, 2.7 and 2.1-fold higher for BR-FB_HCD_ and BR-P_HCD_ in relation to the BR-B run. Overall, these results show that the CDE was successfully overcome by the designed feeding strategies on the bioreactor scale, and the increase in productivity for the developed HCD processes was maintained in the scale-up. However, the bioreactor operating parameters could potentially be further optimized so as to maintain cells in a more efficient metabolic state and maximize production.

### 3.4. IAV Production and Harvest Using the TFDF System

**Figure 7:**
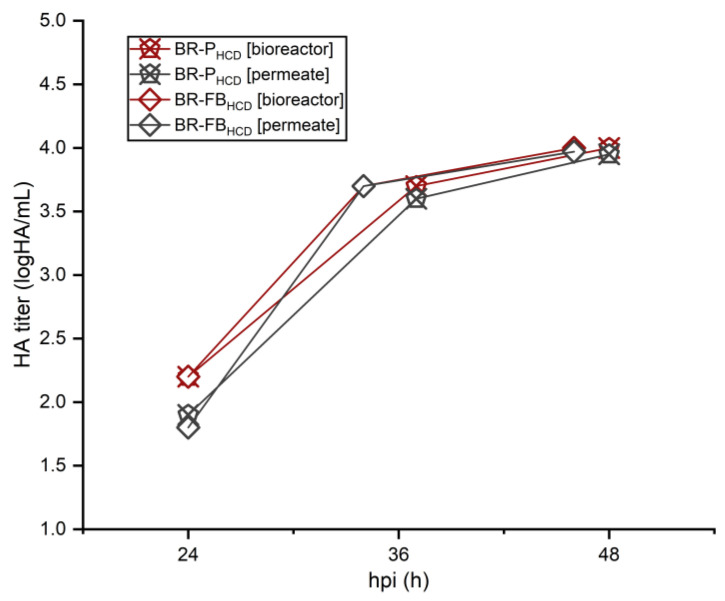
HA content in the bioreactor broth (red) and in the permeate (gray) for 3 L bioreactor cultures of HEK293SF cells operated in fed-batch (BR-FB_HCD_) and perfusion (BR-P_HCD_).

In this study, a TFDF membrane module was used as the cell retention device for the perfusion runs, as well as the harvest unit for all bioreactor runs. To evaluate virus retention during the course of the perfusion-based runs, samples from the bioreactor content and permeate were routinely taken and quantified. HA content for different time points after infection is presented in Figure 7. For both perfusion-based bioreactor runs, BR-P_HCD_ and BR-FB_HCD_, HA content from the bioreactor and permeate samples were the same or remained within the assay error (0.15 logHAU/mL) throughout the infection phase, indicating that little to no virus was being retained in the bioreactor or being lost inside the membrane module. As to the perfusion bioreactor, BR-P_HCD_, samples taken at 48 hpi were quantified by TCID50, and infectious titers for the bioreactor broth and permeate were comparable, respectively, at 1.14 × 10^8^ IVP/mL and 1.12 × 10^8^ IVP/mL. A final harvest step was carried out for all three bioreactor runs, using the same membrane module that was employed during the perfusion phase for the BR-P_HCD_ and BR-FB_HCD_ runs, while using a new membrane for the BR-B batch run. As the harvest process through the TFDF system comprises a short diafiltration step, an increase in the harvest volume of 15–20% is expected. The total virus yield in the bioreactor (previous to the harvest step) and the total yield in the harvested volume are presented in Table 3. Total infectious yield and total HA content indicate that no virus retention nor virus loss occurred during the harvesting step for all three bioreactor runs.

## 4. Discussion

### 4.1. Small-Scale Characterization of the CDE

Previous studies have shown that the CDE observed in HCD virus production processes could be successfully overcome with adequate feeding strategies, and that it could be related to nutrient limitation and inhibition by toxic by-products [17,27,28]. Metabolic flux analysis of suspension HEK293 cell cultures producing adenovirus in low-cell-density and HCD processes operated in batch and perfusion indicated that cell-specific virus productivity was maintained when feeding strategies were able to keep metabolic flux distribution profiles of HCD cultures at similar levels to low-cell-density processes [18]. In the present study, the metabolic state of cells in different stages of the process was estimated based on the average specific uptake/production rates for main metabolites, which were calculated through basic mass balance equations (Equation (1)). The sharp decrease in *CSVY* (Figure 1B) observed for ST-B_M_ and ST-P_H_ conditions was associated with reduced values of *q_GLN_* and *q_NH4_* before and after infection, which are most probably related to the low residual concentration of glutamine around the TOI for those cultures. Regarding glucose metabolism, although the residual concentration of the sugar was much lower for those cultures around the TOI, the difference between the values of *q_GLUC_* and *q_LAC_* was less pronounced for those in relation to the batch control, notably after infection (Figure 1A). These results suggest that glutamine metabolism, both before and after infection, plays an important role in the CDE observed in HEK293SF cell cultures infected at higher cell densities.

### 4.2. Small-Scale Development of HCD Feeding Strategies

In this study, two feeding strategies were developed in small-scale experiments: a hybrid fed-batch with continuous harvest (ST-FB_HCD_) and a low-PR perfusion (ST-P_HCD_). Feeding strategies were designed based on average metabolite rates to replenish consumed nutrients without overfeeding. Both feeding strategies were able to maintain the *CSVY* of cultures infected at 9 × 10^6^ cells/mL at comparable levels to that of batch cultures infected at 2 × 10^6^ cells/mL (Figure 3). While average *q_GLN_* and *q_NH4_* were at similar levels between the HCD cultures and the batch control both before and after infection, *q_GLUC_* was around 2-fold lower for HCD cultures in the 48 h prior to infection (Figure 2). These results support the conclusion that, although glucose supply remains critical, glutamine metabolism has an important part in IAV production in HEK293SF cells.

The molar lactate to glucose ratio (*Y_LAC/GLUC_*) was lower for both HCD strategies and, while this might indicate a more efficient use of glucose—which was also reported by Henry et al. [18] in HCD cultures producing adenoviral vectors—it may also be related to the accumulation of lactate in the culture media, which has been shown to play a role in shifting the metabolism towards lactate consumption [46]. In fact, the average *q_LAC_* was negative on the 48 h prior to infection for ST-FB_HCD_ cultures, at around −0.08 pmol/cell/day, indicating that lactate was being consumed by the cells (Figure 2).

### 4.3. Scale Up to 3 L STR Bioreactor

The scale-up of the developed HCD feeding strategies was performed in a 3 L STR bioreactor employing a TFDF system as a cell retention device. While growth and viability profiles were similar between the two production scales during the growth phase for all three operation modes (batch, hybrid fed-batch, and perfusion), all three bioreactor cultures showed a sharp decrease in growth rate after infection (Figure 5 and Figure 6). As infected cells are reportedly more susceptible to shear stress [47], this reduction in cell growth after infection could be related to higher shear stress for the bioreactor cultures. On the other hand, differences in the operation and control, such as oxygen transfer and pH control, could also result in variations in metabolism and growth [45]. In fact, while glutamine consumption (*q_GLN_*) and its molar ratio to ammonium (*Y_NH4/GLN_*) were overall comparable for the same feeding strategies in the different production scales (50 mL shake-tubes and 3 L STR bioreactor), glucose uptake and lactate production were much higher for bioreactor cultures. Furthermore, glucose metabolism was less efficient both before and after infection for bioreactor cultures, with *Y_LAC/GLUC_* values around 5-fold higher (Table 2), which led to increased lactate accumulation. These differences in metabolism could be related to the bioreactor operation parameters, notably the oxygen supply. A study performed by Zupke et al. [45] on hybridoma cells has shown that the flux of pyruvate to the TCA cycle greatly decreases in cells growing at low dissolved oxygen concentrations, resulting in higher glucose uptake and lactate accumulation. Nonetheless, virus titers and productivity remained comparable between the different production scales for all three operation modes (Figure 3). Both developed HCD feeding strategies were able to overcome the CDE when applied at the 3 L bioreactor scale, which resulted in an overall increase in the process productivity, notably for the hybrid fed-batch operation (1.7-fold higher *Y/M* and 2.7-fold higher *STY* when compared to the batch run). Other studies on the intensification of IAV production in different cell lines also reported successfully overcoming the CDE in HCD cultures through the development of perfusion-based feeding strategies employing higher perfusion rates [27,28,29,30].

With respect to the virus titers obtained, the HA contents attained with the two HCD processes developed are comparable to what was reported with other suspension cell lines [27,28,29] but lower than what has been reported for suspension MDCK cells [48], known to be one of the preferable hosts for influenza production. While the infectious titers achieved in the present study are lower than what was previously reported for other HCD processes for IAV production [20,27,29,30], this discrepancy is most likely related to the assay conditions, such as temperature shift, cell line, and use of crystal violet stain or specific antibodies. In fact, a study performed using the same cell line for IAV production reported much higher infectious titers for samples with similar or lower HA content [20]. Therefore, direct comparisons of virus titers between different studies were avoided.

While the concentration of host cell DNA and proteins—the main contaminants in cell culture-based vaccine preparations—was not assessed for the different processes developed in the present study, it is expected that higher cell densities at infection result in higher concentrations of those contaminants in the produced material. Therefore, the development of robust downstream processes is critical to support cell culture-based influenza vaccines. A robust and scalable process for the purification of influenza viruses produced in HEK293SF cells is described [49], reporting up to 96.5% and 99.7% removal of host cell DNA and proteins, respectively.

### 4.4. IAV Production and Harvest Using the TFDF System

No virus retention was observed on all three bioreactor runs employing the TFDF system, neither during the production phase nor during the harvesting step (Figure 7 and Table 3). Other membrane-based cell retention devices, notably the ATF system, have also been employed for IAV production, but virus retention due to progressive membrane clogging was reported for membrane pores sizes < 0.5 µm [27,28]. As for all membrane-based cell retention devices, shear stress might be an issue with the TFDF system, but optimization of culture parameters such as the recirculation rate can alleviate its effects. In this study, no detrimental effect of shear stress over virus production was observed, neither during the infection phase nor during the final harvest step. Other cell retention devices, notably acoustic settlers and inclined settlers, have also been applied for virus production [20,29,30], with the advantage of no virus retention and low shear stress, the latter being critical during the infection phase as infected cells tend to be more shear-sensitive [30,47]. However, poor scalability due to heat exchange limitations is the main drawback of this kind of device. The TFDF system, on the other hand, is scalable and could support large-scale influenza vaccine manufacture: as of now, the system is available for operations of up to 2000 L [50], which is consistent with large-scale production of cell culture-based viral vaccines.

## 5. Conclusions

The present study demonstrated that perfusion-based feeding strategies that minimize media handling and consumption are capable of overcoming metabolic limitations when applied to HCD processes for IAV production. Suspension HEK293SF cultures operated either in fed-batch with continuous harvest or perfusion can be infected at up to 9 × 10^6^ cells/mL without significant loss in *CSVY*, which resulted in an overall increase in the process productivity. We have also demonstrated that the estimation of average uptake/production rates for main metabolites is a useful approach for designing and evaluating feeding strategies, notably during the scaling up of processes, as differences in operation and control of culture parameters can significantly alter cell metabolism. This work also showed that TFDF membranes are suitable for large-scale influenza virus production as the system is scalable, and no virus retention nor loss was observed during the production and harvest steps. In order to validate the production process, further characterization of the produced virus, as well as the evaluation of the efficacy of the vaccine preparation, should be performed. As HEK293 cells have been shown to be a safe and well-established cell platform, the strategies and processes developed in this study could possibly be applied to intensify the production of other viral vaccines and vectors using this cell substrate. Future work will explore the relation between specific uptake/production rates and online signals, such as capacitance, fluorescence, and specific respiration rate, for online assessment of the metabolic state of cells.

## Figures and Tables

**Table 1:** Summary of culture conditions and results.

Run Code	ST-B	ST-B_M_	ST-P_H_	ST-FB_HCD_	ST-P_HCD_	BR-B	BR-FB_HCD_	BR-P_HCD_
V_R_ (mL)	10	10	10	10	10	1800	2000	2000
Growth phase	Batch	Batch	Perfusion ^c^ (0.5 VVD)	Fed-batch	Perfusion ^c^ (0.5 VVD)	Batch	Fed-batch	Perfusion (0.4–0.8 VVD)
Infection phase	Batch	Batch	Perfusion ^c^ (0.5 VVD)	Perfusion ^c^ (0.5 VVD)	Perfusion ^c^ (0.5 VVD)	Batch	Perfusion (0.5 VVD)	Perfusion (0.5 VVD)
VCD_0_ (10^6^ cells/mL)	0.35	0.35	0.35	2	1.5	0.5	2	1.5
VCD_TOI_ (10^6^ cells/mL)	2	4	13	8–10	8–10	1.6	8.5	8.6
VCD_MAX_ (10^6^ cells/mL) ^a^	3.2 ± 0.16	8.1 ± 0.4	20.5 ± 2.16	14.3 ± 0.08	15.0 ± 0.05	2.4 ± 0.16	10.1 ± 0.42	10 ± 0.5
HA_TOH_ (logHAU/mL)	3.5	3.1	3.65	4.11	4.26	3.7	4.2	4
TCID_TOH_ (10^8^ IVP/mL) ^a^	0.86 ± 0.15	-	-	5.28 ± 0.07	6.75 ± 1.5	0.53 ± 0.1	1.01	1.14 ± 0.21
HA_Tot_ (HAU) ^b^	(3.20 ± 0.2) × 10^4^	(1.28 ± 0.3) × 10^4^	(5.44 ± 0.1) × 10^4^	(1.30 ± 0.01) × 10^5^	(1.86 ± 0.01) × 10^5^	8.1 × 10^6^	3.4 × 10^7^	2.4 × 10^7^
Y/M (10^6^ HAU/L) ^b^	3.20 ± 0.06	1.28 ± 0.2	1.53 ± 0.08	6.51 ± 0.04	6.45 ± 0.06	4.53	7.81	2.98
CSVY (HAU/10^6^ cell) ^b^	917 ± 12	122 ± 17	209 ± 35	834 ± 2	1129 ± 7	1311	1268	1220
CSVY_TCID_ (IVP/cell) ^b^	24.7 ± 4	-	-	-	-	15	-	14
STY (10^6^ HAU/L/day) ^b^	0.64 ± 0.01	0.18 ± 0.01	0.68 ± 0.01	2.60 ± 0.03	3.72 ± 0.02	1.1	3	2.3
Total media	1 × V_R_	1 × V_R_	4 × V_R_	2 × V_R_	2.9 × V_R_	1 × V_R_	2.2 × V_R_	4 × V_R_

Abbreviations: VCD_0_, initial viable cell density; VCD_TOI_, viable cell density at time of infection; VCD_MAX_, maximum viable cell density; HA_TOH_, HA content at time of harvest; TCID_TOH_, infectious virus titer at time of harvest; HA_Tot_, total yield based on HA content; Y/M, yield on media based on HA content; CSVY, cell-specific virus yield based on HA content; CSVY_TCID_, cell-specific infectious virus yield; STY, space–time yield based on HA content; V_R_, reactor working volume. ^a^ Standard deviation was calculated based on the biological duplicates for ST cultures and based on duplicates for the same time point for BR cultures; ^b^ Standard deviation for productivity parameters was calculated based on biological duplicates for ST cultures; ^c^ Perfusion operations were performed semi-continuously (pseudo-perfusion) for all shake-tube experiments.

**Table 2:** Average values of *Y_LAC/GLUC_* and *Y_NH4/GLN_* in the 48 h prior to the infection (−48 hpi) and 24 h following the infection (24 hpi) for all cultures.

		ST-B	ST-B_M_	ST-P_H_	ST-FB_HCD_	ST-P_HCD_	BR-B	BR-FB_HCD_	BR-P_HCD_
*Y_LAC/GLUC_*	−48 hpi	1.75 ± 0.4	0.2 ± 0.04	0.57± 0.002	0.06 ± 0.00	0.73 ± 0.02	1.58	0.78	1.29
	24 hpi	0.31 ± 0.04	0.87 ± 0.12	0.59 ± 0.15	0.32 ± 0.05	0.31 ± 0.01	1.64	1.24	1.46
*Y_NH4/GLN_*	−48 hpi	0.51 ± 0.05	0.07 ± 0.00	0.29 ± 0.1	0.22 ± 0.01	0.31 ± 0.001	0.53	0.01	0.2
	24 hpi	0.09 ± 0.03	2.97 ± 0.44	0.83 ± 0.01	0.06 ± 0.01	0.16 ± 0.001	0.06	0.47	0.12

**Table 3:** Volume and virus content before and after harvest through a TFDF membrane module.

	Volume (mL)		Virus Yield (logHAU)		Virus Yield (IVP)	
	Bioreactor (TOH)	TFDF Harvest	Bioreactor (TOH)	TFDF Harvest	Bioreactor (TOH)	TFDF Harvest
BR-B	1720	1950	6.89	6.95	7.9 × 10^10^	5.66 × 10^10^
BR-FB_HCD_	2100	2510	7.48	7.49	2.1 × 10^11^	2.3 × 10^11^
BR-P_HCD_	1900	2150	7.29	7.18	2.2 × 10^11^	1.7 × 10^11^

## Data Availability

Additional data are available on request from the corresponding author.

## Supplementary Materials

The following supporting information can be downloaded at: https://www.mdpi.com/article/10.3390/vaccines11121819/s1, Figure S1: Nutrient and metabolite profile for 50 mL shake-tube cultures of HEK293SF cells for influenza virus production, consisting of: (A) low-cell-density batch (ST-B, infected at 2 × 10^6^ cells/mL), (B) medium-cell-density batch (ST-B_M_, infected at 4 × 10^6^ cells/mL) and (C) high-cell-density 0.5 VVD pseudo-perfusion (ST-P_H_, infected at 13 × 10^6^ cells/mL); Figure S2: Nutrient and metabolite profile for 50 mL shake-tube cultures of HEK293SF cells for influenza virus production, consisting of: (A) pseudo-perfusion (ST-P_HCD_, infected at 9.5 × 10^6^ cells/mL), and (B) hybrid fed-batch/pseudo-perfusion (ST-FB_HCD_, infected at 8.5 × 10^6^ cells/mL); Figure S3: Nutrient and metabolite profile for 3 L STR bioreactor cultures of HEK293SF cells for influenza virus production, consisting of: (A) low-cell-density batch (BR-B, infected at 2 × 10^6^ cells/mL), (B) perfusion (BR-P_HCD_, infected at 8.5 × 10^6^ cells/mL), and (C) hybrid fed-batch/perfusion (BR-FB_HCD_, infected at 8.5 × 10^6^ cells/mL).

## Abbreviations

TFDF, tangential flow depth filtration; ATF, alternating tangential flow filtration; TFF, tangential flow filtration; CDE, cell density effect; IAV, influenza A virus; HIFB, high inoculum fed-batch; HCD, high cell density; hpi, hours post-infection; PR, perfusion rate; CSVY, cell-specific virus yield; Y/M, yield on media; STY, space–time yield; VVD, vessel volume per day; MOI, multiplicity of infection; VCD, viable cell density; TOI, time of infection; TOH, time of harvest; HA, hemagglutinin; IVP, infectious virus particles; q, average cell specific uptake/production rate; GLUC, glucose; LAC, lactate; GLN, glutamine; GLU, glutamate; NH4, ammonium.
