# Supplementary material for: Intensified Influenza Virus Production in Suspension HEK293SF Cell Cultures Operated in Fed-Batch or Perfusion with Continuous Harvest
Source: Vaccines (Basel). 2023 Dec 5;11(12):1819. doi: 10.3390/vaccines11121819 (PMC10747379; doi:10.3390/vaccines11121819)
Supplement: Supplementary file 1 [file vaccines-11-01819-s001.zip › vaccines-2707850-supplementary.pdf]

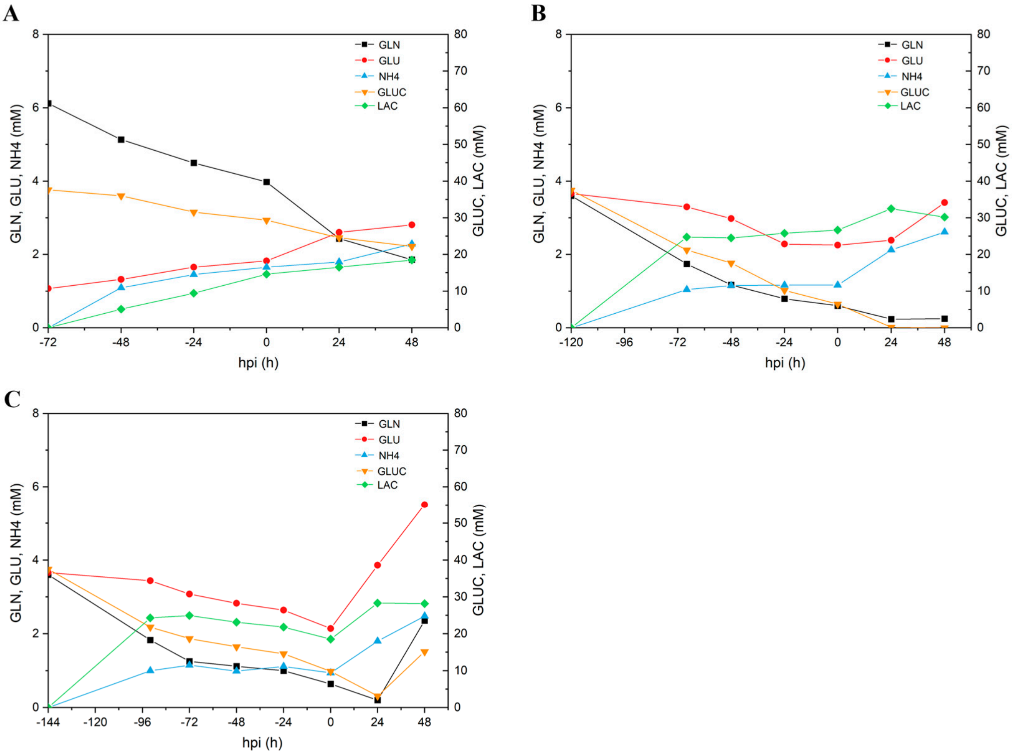

Figure S1 Nutrient and metabolite profile for 50 mL shake-tube cultures of HEK293SF cells for influenza virus production, consisting of: (A) low cell density batch (ST-B, infected at 2 × 10^6 cells/mL), (B) medium cell density batch (ST-B_M, infected at 4 × 10^6 cells/mL) and (C) high cell density 0.5 VVD pseudo-perfusion (ST-P_H, infected at 13 × 10^6 cells/mL). As supernatant samples were taken before media exchange or feeding, the concentration of nutrients/metabolites immediately after media exchange or feeding are not presented in the graphs.

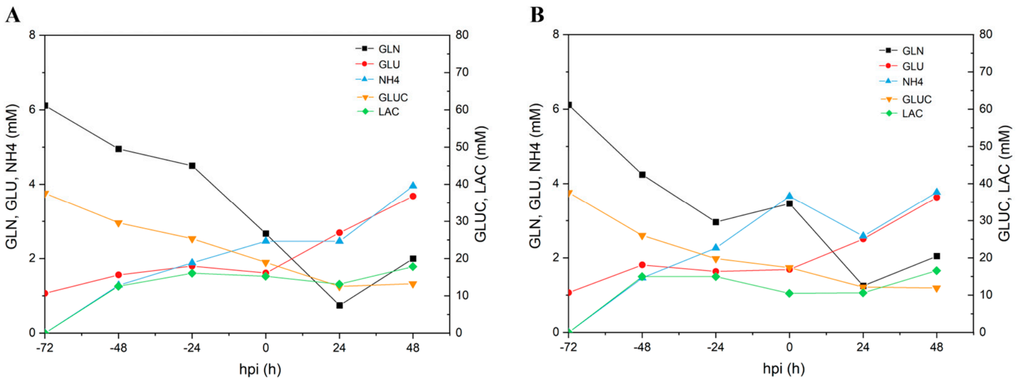

Figure S2 Nutrient and metabolite profile for 50 mL shake-tube cultures of HEK293SF cells for influenza virus production, consisting of: (A) pseudo-perfusion (ST-P_{HCD}, infected at 9.5 × 10^6 cells/mL), and (B) hybrid fed-batch/pseudo-perfusion (ST-FB_{HCD}, infected at 8.5 × 10^6 cells/mL).

As supernatant samples were taken before media exchange or feeding, the concentration of nutrients/metabolites immediately after media exchange or feeding are not presented in the graph.

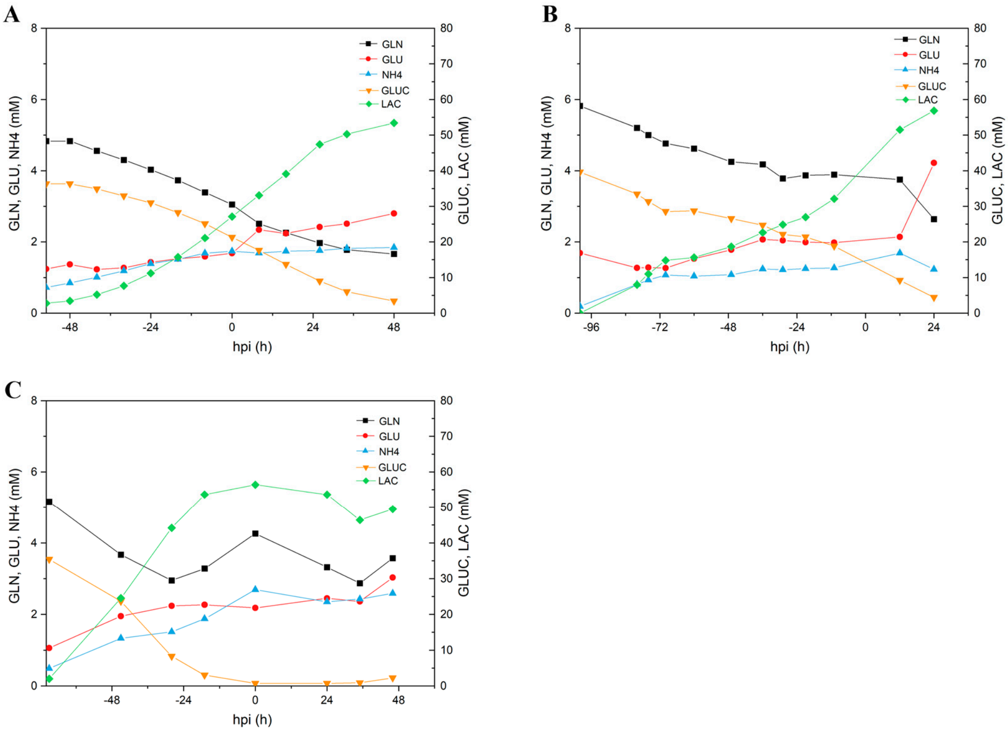

Figure S3 Nutrient and metabolite profile for 3 L STR bioreactor cultures of HEK293SF cells for influenza virus production, consisting of: (A) low cell density batch (BR-B, infected at 2×10^6 cells/mL), (B) perfusion (BR-P_{HCD}, infected at 8.5×10^6 cells/mL), and (C) hybrid fed-batch/perfusion (BR-FB_{HCD}, infected at 8.5×10^6 cells/mL)
